# Role of convolutional features and machine learning for predicting student academic performance from MOODLE data

**DOI:** 10.1371/journal.pone.0293061

**Published:** 2023-11-08

**Authors:** Nihal Abuzinadah, Muhammad Umer, Abid Ishaq, Abdullah Al Hejaili, Shtwai Alsubai, Ala’ Abdulmajid Eshmawi, Abdullah Mohamed, Imran Ashraf

**Affiliations:** 1 Department of Computer Science, Faculty of Computing and Information Technology, King Abdulaziz University, Jeddah, Saudi Arabia; 2 Department of Computer Science & Information Technology, The Islamia University of Bahawalpur, Bahawalpur, Pakistan; 3 Faculty of Computers & Information Technology, Computer Science Department, University of Tabuk, Tabuk, Saudi Arabia; 4 Department of Computer Science, College of Computer Engineering and Sciences in Al-Kharj, Prince Sattam bin Abdulaziz University, Al-Kharj, Saudi Arabia; 5 Department of Cybersecurity, College of Computer Science and Engineering, University of Jeddah, Jeddah, Saudia Arabia; 6 Research Centre, Future University in Egypt, New Cairo, Egypt; 7 Information and Communication Engineering, Yeungnam University, Gyeongsan, Korea; Menoufia University, EGYPT

## Abstract

Predicting student performance automatically is of utmost importance, due to the substantial volume of data within educational databases. Educational data mining (EDM) devises techniques to uncover insights from data originating in educational settings. Artificial intelligence (AI) can mine educational data to predict student performance and provide measures to help students avoid failing and learn better. Learning platforms complement traditional learning settings by analyzing student performance, which can help reduce the chance of student failure. Existing methods for student performance prediction in educational data mining faced challenges such as limited accuracy, imbalanced data, and difficulties in feature engineering. These issues hindered effective adaptability and generalization across diverse educational contexts. This study proposes a machine learning-based system with deep convoluted features for the prediction of students’ academic performance. The proposed framework is employed to predict student academic performance using balanced as well as, imbalanced datasets using the synthetic minority oversampling technique (SMOTE). In addition, the performance is also evaluated using the original and deep convoluted features. Experimental results indicate that the use of deep convoluted features provides improved prediction accuracy compared to original features. Results obtained using the extra tree classifier with convoluted features show the highest classification accuracy of 99.9%. In comparison with the state-of-the-art approaches, the proposed approach achieved higher performance. This research introduces a powerful AI-driven system for student performance prediction, offering substantial advancements in accuracy compared to existing approaches.

## Introduction

Particularly in the context of the COVID-19 pandemic, which brought traditional educational systems to a halt, the rapid advancement of technology and the widespread adoption of technology-assisted educational platforms opened the doors to new paradigms for the educational systems [1, 2]. Online platforms can keep an eye on students’ activities and gather information that can be used to study students’ academic performance and take necessary actions promptly for the betterment of the students. Thus, preventive and timely actions can reduce the possibility of student failure and enhance the performance of academic institutions. An emerging area of research called educational data mining (EDM) looks at educational data to perform various academic tasks. EDM is most frequently used to predict students’ academic performance. In data mining, the analysis and the interpretation of student academic performance are considered suitable analysis, evaluation, and assessment techniques. Students are the essential component for a country’s socioeconomic success in today’s knowledge-based economy. Therefore, it is important to maintain students’ academic progress [3]. Higher education institutions (HEIs) use a wide range of information and communication technology (ICT) based learning techniques to facilitate teaching and learning and to impart knowledge to their students. Such methods use a variety of learning environments [4]. For auditing and recovery purposes, these platforms also keep a record of students and their interactions with the Platform.

Students at higher education institutions access the internet, learning management systems (LMS), and other similar kinds of platforms. these digital learning environments can store data that can be viewed at later stages. LMS [5, 6], student logs (Moodle), and video interactions [7] are often utilized in educational settings, and their statistics are used to evaluate students’ academic performance. The adoption of these systems in educational institutions generates a lot of data that may be used for further research to examine factors related to students’ performance. The factors that influence students’ performance can be further analyzed to help take necessary actions. The analysis of the student academic performance data helps in improving the teaching quality and academic performance as well [8]. However, handling such a massive amount of data is a difficult and time-consuming task, if we handle it manually. In the age of technology, machine learning-based systems open new doors for student academic performance prediction and EDM can handle the problems efficiently [9].

Researchers have undertaken the task of predicting students’ marks and grades, with the assessment relying heavily on the student’s prior educational records. Using these forecasts, students displaying weaker grades and lower marks will be identified, and tailored support and attention will be provided to help them excel in their examinations. The authors applied a regression model to predict the marks of students and a decision tree model to classify the grades of students [10]. Researchers applied emotional factors on online platforms to predict the educational performance of students [11]. Authors used the fuzzy logic model to predict student performance [12].

A large number of studies are available that use EDM for student academic performance prediction [13, 14]. However, demographic data was the main focus of these studies, and predictions were made based on activities carried out in the online environment. However, only a few researchers [15, 16] examined the video interactions of students in a video-assisted course. Using educational data mining, this study makes use of various online video learning sources such as Moodle, eDify, and SIS data to access the performance of the students in the online video learning environments. Analyzing these videos, the teaching and learning process can be improved [17]. In this regard, this work makes the following contributions.

- This study proposes an ensemble model that utilizes convoluted features from a customized Convolutional Neural Network (CNN) model to train machine learning models. The model is used for predicting students’ academic performance using academic features, video interactions, and student activities.
- Experiments are performed to analyze the performance of the proposed approach from two perspectives. First, performance comparison with the imbalanced dataset versus the SMOTE-balanced dataset. Secondly, performance is compared against the original features versus convoluted features.
- For performance comparison, several machine learning models are used including stochastic gradient descent (SGD), gradient boosting machine (GBM), random forest (RF), logistic regression (LR), extra tree classifier (ETC), and Gaussian Naive Bayes (GNB). Also, the performance is compared with several state-of-the-art models.

The organization of this paper is as follows. Section 2 briefly describes the most recent literature related to students’ academic performance prediction. Section 3 describes the proposed methodology while the results are presented in Section 4. Finally, the conclusion is given in Section 5.

## Literature review

In recent years, EDM has become an effective and well-known technique for uncovering the hidden patterns lying inside educational data, predicting academic performance, and enhancing the learning and teaching environment. E-learning systems have been effectively analyzed in several EDM studies. While some research works classify educational data, others tried to predict student performance. To enhance students’ performance in various academic tasks, numerous studies have been carried out. This section provides a brief overview of the past related works.

Similar to the use of machine learning models in other domains [18–20], predicting student academic performance has also adopted machine learning models. Using the grades from midterm exams as the source data, Mustafa Yağci [21] proposed a machine learning-based approach for predicting undergraduate students’ final test results. For predicting students’ final exam grades, multiple machine learning methods including RF, Naive Bayes (NB), support vector machine (SVM), LR, and k nearest neighbor (KNN) algorithms were used. The dataset used in this study is taken from the Turkish State University, in which 1854 students took a Turkish-I course in the fall semester of 2019-2020. The results of the study show that the proposed model achieved an accuracy value of 70% to 75%. A machine learning-based approach was presented by Rebai et al. in [22] to find the factors that affect students’ academic performance in schools and the relationship among these factors. The results show that class size, school size, gender proportions, competition, and parental pressure affect the student’s academic performance directly. Furthermore, the results of RF showed that the number of students in the school and the girl’s percentage had a direct and large impact on the model’s predicted accuracy.

Waheed et al. [23] proposed an artificial neural network (ANN) system based which employed student records related to their LMS navigation. Demographics and student clickstream activities were found to have a substantial impact on student performance. Students who completed their courses performed better. In addition, the authors concluded that deep learning models could be the best choice for student performance prediction. Ayon et al. [24] proposed artificial intelligence (AI)-based algorithms for predicting academic achievements and recommending appropriate study plans to improve student performance. Sophisticated deep learning algorithms and ANN-based models are two models developed in their study. Experimental results showed an accuracy of 97.02% and study planner models demonstrated an accuracy of 99.8% on training datasets, and 92.94% and 87.65% on test datasets respectively. To predict and optimize the number of test administrations, Shin et al. [25] used clustering approaches and proposed a deep learning system. They collected 10107 first graders’ math performance data for the period of 2017 to 2018 academic years. Their best model produced good results, with an accuracy of 90%. Furthermore, the clustering method provided interpretable insights into the relationship between test administration decisions and student performance profiles.

For the prediction of students’ academic performance at school and home an improved conditional generative adversarial network (CGAN) is proposed by Samina et al. [26]. They also coupled the CGAN with the deep-layered SVM. The dataset used in this study is very small in size; improved CGAN is used to create the synthetic data samples. The authors also compared the results with and without CGAN use. The proposed approach shows better results on the combination of home and school tutoring and shows that both have a great impact on the student’s academic performance. Saman et al. [27] used the various EDM methods for accessing the performance as well and they carried out the analysis of different features that have a significant effect on students’ academic performance. Several classification techniques, such as RF, decision tree (DT), SGD classifier, AdaBoost classifier, SVM classifier, and LR classifier were used. The results showed that RF outperformed other classifiers and achieved an accuracy of 98%.

For the prediction of student academic performance a deep learning-based system is proposed by Yousafzai et al [28]. The authors utilize the BiLSTM model to obtain better results. The quality of BiLSTM to handle the superior sequence learning with attention mechanism gives the best results in comparison with the other state-of-the-art models. Experimental results showed an accuracy score of 90.16%. To detect the student’s confusion level at the massive open online course (MOOC) platform Daghriri et al. [29] used electroencephalogram (EEG) data. The authors proposed a novel engineering approach for improving the efficacy of machine learning models by using probability-based features (PBF). For the machine learning model training, the PBF techniques use the probabilistic output of RF and GBM as feature vectors. The study’s results indicate that using the PBF technique on EEG data can achieve 100% accuracy in spotting confused students. Table 1 summarizes the work of past studies, which shows that those studies use machine learning or deep learning models for prediction. However, this study uses CNN features in combination with machine learning models to get deeper insight into the dataset.

**Table 1:** Detailed description of the dataset.

Ref.	Prediction Approach	Considered Factors	Contributions
[21]	Machine learning models (RF, NB, SVM, LR, KNN)	Midterm exam grades, Turkish-I course	Predicting undergraduate students’ final test results with 70% to 75% accuracy
[22]	Machine learning models (RF)	Class size, school size, gender proportions, competition, parental pressure	Identifying factors affecting students’ academic performance
[23]	Artificial Neural Network (ANN)	Demographics, clickstream activities	Emphasizing the impact of demographics and clickstream activities
[24]	Deep learning algorithms, ANN	Student performance factors	Recommending study plans and achieving high accuracy
[25]	Clustering approaches, deep learning	10,107 first graders performance data	Optimizing test administrations and clustering insights
[26]	Conditional Generative Adversarial Network (CGAN), SVM	Home and school tutoring	Enhanced CGAN-SVM approach for school and home performance
[27]	Various EDM methods, machine learning models (RF, DT, SGD, AdaBoost, SVM, LR)	Various academic features	Analysis of features affecting academic performance
[28]	BiLSTM model	student grade prediction dataset 33 attributes with 1044 records	BiLSTM with attention mechanism for performance prediction
[29]	EEG data, machine learning models (RF, GBM)	EEG data	Detection of student confusion using EEG data

## Materials and methods

**Fig 1:**
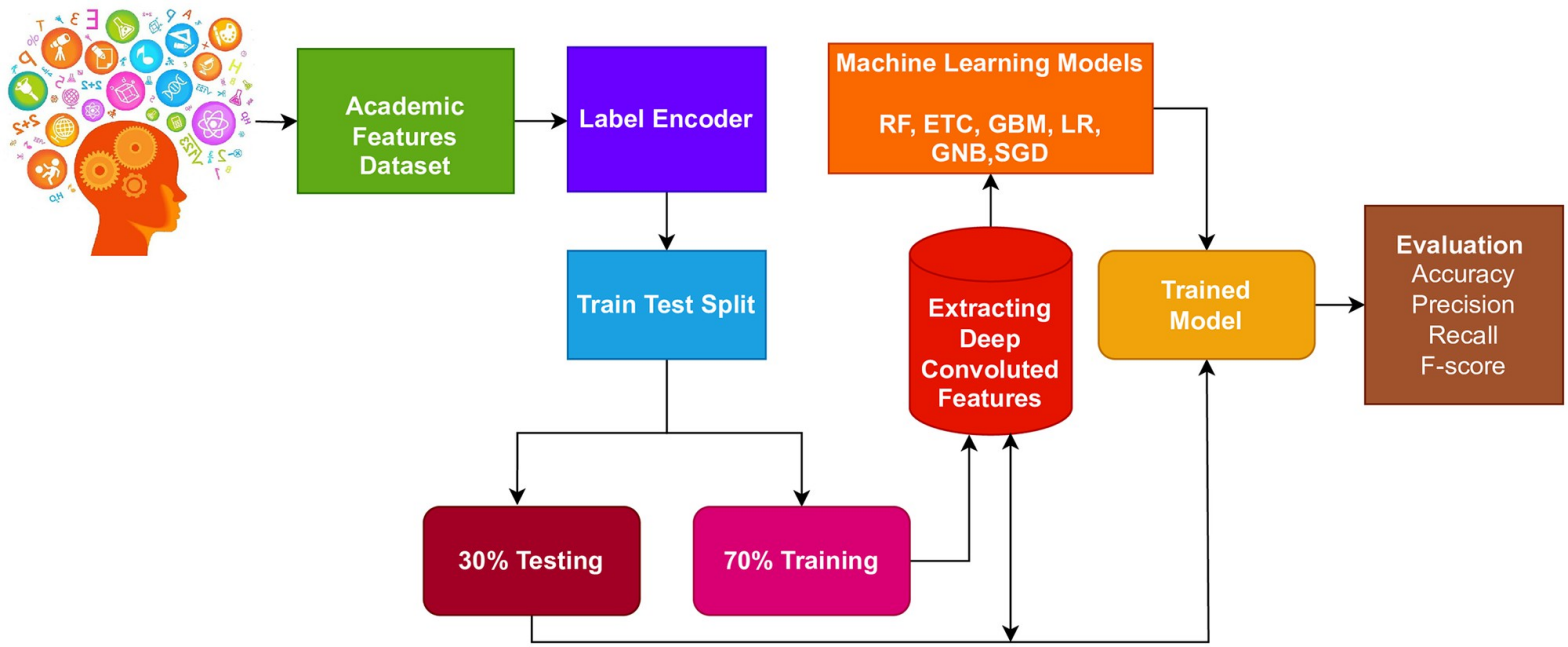
Workflow diagram of the adopted methodology.

In this section, the proposed approach, the dataset used in this study, and the steps followed for the proposed approach are discussed. Fig 1 shows the workflow of the proposed approach.

### Overview

Firstly, the data collection phase involved utilizing a dataset obtained from eDify Moodle, which consists of features associated with students’ academic performance. ‘Moodle’ is a popular open-source learning management system (LMS) or e-learning platform. It was developed by Martin Dougiamas in 2002 with a focus on creating an online learning environment that promotes collaboration and interaction between educators and learners. ‘eDify’ is a mobile application for this purpose. The features from the data were extracted and subjected to preprocessing using a label encoder to convert categorical data into a numeric format. Subsequently, the processed features were divided into a training set comprising 70% of the data and a testing set comprising the remaining 30% using sklearn’s train-test validation. The machine learning model was then trained on the training set using deep convolutional features.

**Fig 2:**
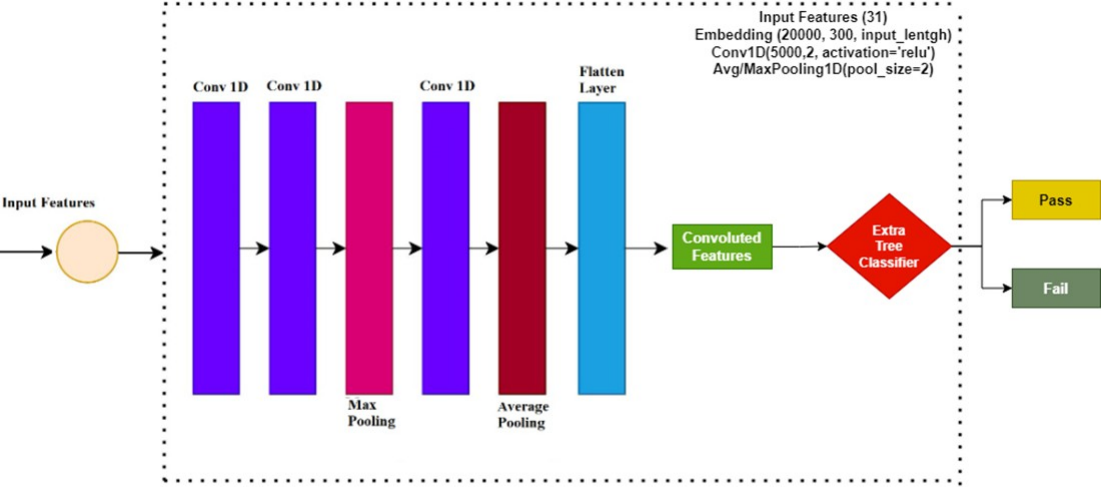
Architectural diagram of the proposed methodology.

This study introduces an ensemble model that combines deep convolutional features with a machine-learning model for classification purposes. The architecture of the proposed ensemble model is depicted in Fig 2. To predict student academic performance, this proposed approach employs the ETC machine learning model, deviating from the use of manually engineered features. Instead, a customized convolutional neural network (CNN) is employed to extract relevant and significant features from the dataset. These extracted features are subsequently utilized as input for training the ETC model. Then the trained model is evaluated in terms of accuracy, recall, precision, and F1 score.

### Dataset

**Fig 3:**
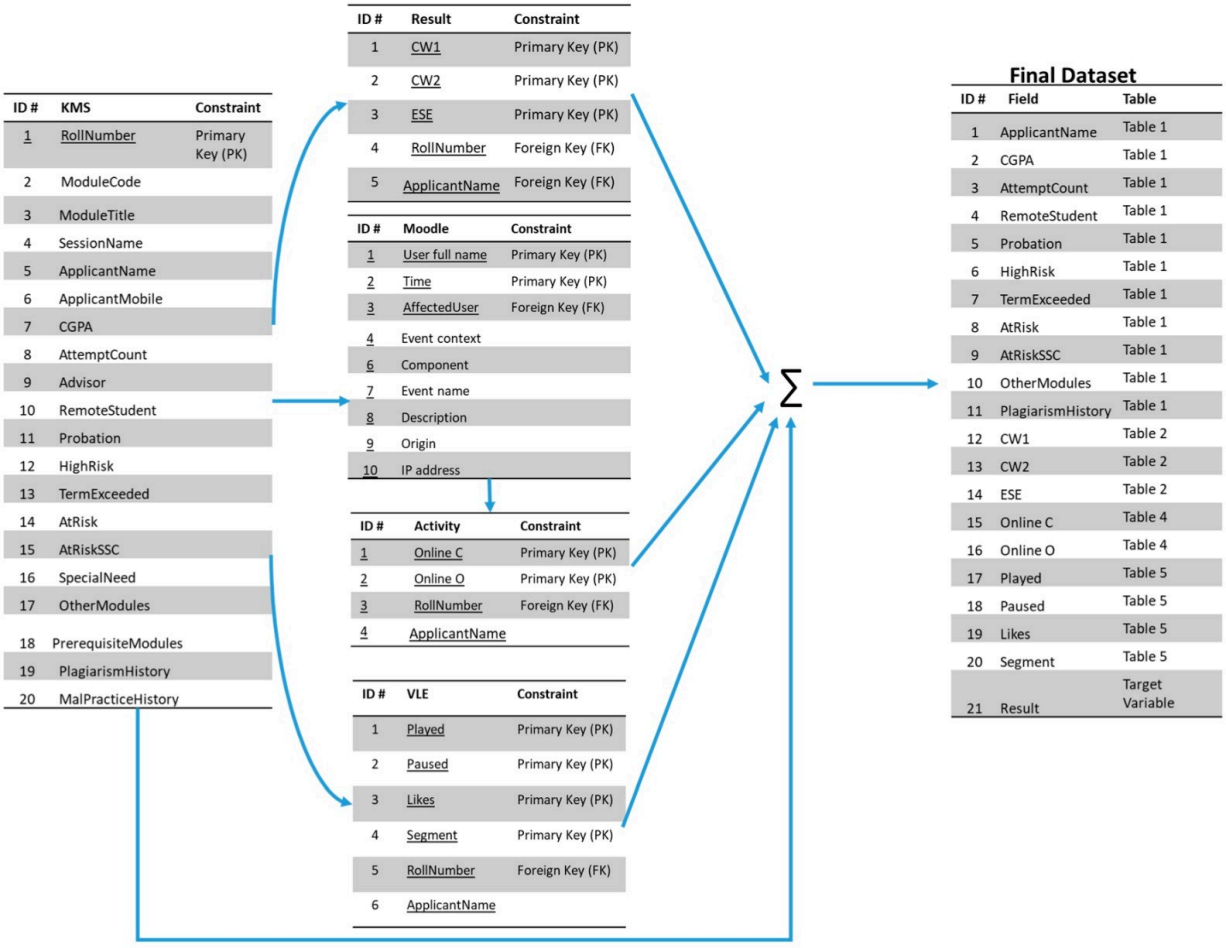
Complete mapping of the dataset.

The dataset used in this study is obtained from Hasan et al. [30]. The dataset contains different features obtained from MOODLE. A visual presentation of the dataset along with the relationship between its attributes is shown in Fig 3. The final data sets constituted three categories: students’ academic information, students’ video interaction, and students’ activity. From the SIS the student’s academic information data was extracted, eDify data is used for the data of student’s video interactions, and the MOODLE platform is used to extract the students’ activity data. After that, mapping resulted in a final dataset with 326 instances and 21 features. These features are “Applicant Name”, “Attempt Count”, “Prohibition”, “CGPA”, “Remote Student”, “High Risk”, “At Risk”, “Term Exceeded”, “At Risk SSC”, “Plagiarism history”, “CW1”, “Other Modules”, “CW2”, “Online c”, “ESE”, “Online O”, “Paused”, “Played”, “Likes”, “Segment” and “Result”.

A description of the final dataset is given in Table 2. The dataset consists of a total of 326 instances with the target class result as ‘pass’ and ‘fail’. Out of these 326 instances, 264 are passed and 62 students belong to the ‘fail’ class. These statistics show that the dataset is highly imbalanced.

**Table 2:** Detailed description of the dataset.

Attribute	Description
ApplicantName	It contains the student’s name. For example “Student 1”
CGPA	It is the CGPA obtained by the student.
AttemptCount	It shows how many attempts are taken for a specific module.
RemoteStudent	It shows whether a student is taking classes remotely or not.
Probation	It represents the backlog of modules to clear.
HighRisk	It shows the risk of failure from a specific subject.
TermExceeded	It is the progression of a student in a degree.
AtRisk	If a student fails previous modules will be at risk.
AtRiskSSC	It shows the student registration at the student success centre, if he has any educational deficiencies,
OtherModules	It shows the registration of a student in other modules for the current semester,
PlagiarismHistory	It describes the previous plagiarism history of the student in any module.
CW1	It is the marks achieved by the student in the first coursework.
CW2	It is the marks achieved by the student in the second coursework.
ESE	It is the end semester examination obtained marks.
Online C	On campus user-performed activities (in minutes),
Online O	Off-campus user-performed activities (in minutes),
Played	How many times a video has been played
Paused	How many times a video has been paused
Segment	How many times a specific part of the video is played using slider
Likes	How many times a video has been liked by the student
Result	Final Result of the student

### Data resampling techniques

The dataset used in this study is the imbalanced dataset. To handle the imbalance dataset problem efficiently a data resampling technique is used. The target classes in the imbalanced dataset have an unequal ratio, where 264 students belong to the pass class and 62 students belong to the ‘fail’ class. The overfitting problem can happen in the case of using the original imbalanced dataset as the model can overfit the majority class. There is a variety of data resampling techniques available to overcome this problem. From the available data resampling approaches, this study adopts SMOTE due to good results and better performance of machine learning models.

Presently varieties of data resampling techniques are available such as SMOTE, adaptive synthetic resampling (ADASYN), etc. These algorithms use different approaches to up-sample the data. In oversampling the number of samples of the minority class is increased to the number of samples of the majority class. In oversampling, the overall number of samples is increased, which creates additional features for model training and it is helpful for the improvement of model accuracy [31, 32]. For oversampling, SMOTE is used in this study. SMOTE is a cutting-edge technique proposed to address the overfitting problem in imbalanced datasets. To create a new minority class sample, the selected samples are evaluated using the KNN at that specific point. Because new instances are formed based on original features, they are identical to the original data. SMOTE is not the best choice for dealing with high-dimensional data because it creates additional noise.

### Machine learning models used for the student academic performance prediction

Presently, a wide range of machine learning algorithms is available for student academic performance classification. This study uses selected machine learning algorithms for that purpose. We used six machine learning algorithms including GBM, RF, ETC, GNB, LR, and SGD. This section of the study gives a brief description of these algorithms.

#### Random forest

RF is a tree-based ensemble classifier; it combines several weak learners and provides extremely accurate predictions. For the training of the different decision trees, RF uses the bootstrap bagging technique. By the subsampling of the training dataset, RF generates these bootstrap samples, where both the dataset (training, testing) is of the same size for the prediction process [33]. The root node attribute identification at each level is a big problem for the development of decision trees.

$$\begin{array}{c} p=mode\{T_{1}\left(y\right),T_{2}\left(y\right),\ldots \ldots T_{m}\left(y\right)\} \end{array}$$ (1)

$$\begin{array}{c} p=mode\{\sum_{m}^{m=1}\left[T_{m}\left(y\right)\right]\} \end{array}$$ (2)

For the prediction process, *T*_1_(*y*), *T*_2_(*y*), *T*_3_(*y*), and *T*_*m*_(*y*) are the number of decision trees, and *p* shows the decision tree’s final decision by majority votes.

#### Gradient boosting machine

For the creation of a powerful prediction model, many weak learners are combined. A loss function and a customized loss function based on the GBM are also used in it. Generic loss function can be handled efficiently with the help of GBM but there is a need for a defined loss mechanism [34]. Logarithmic loss is also used in classification methods, and for regression problems, squared errors can be used. The gradient boosting system does not need to generate a new loss function, each time the boosting method is performed; instead, any differentiable loss function can be applied to the system. To acquire good accuracy from the GBM, several hyperparameters are tweaked.

#### Extra tree classifier

ETC is a renowned ensemble learning model that makes a final decision based on the results of many uncorrelated decision trees [35]. Using training samples, each decision tree in the forest is used for further classification. Multiple uncorrelated decision trees are built from a random sampling of features. Feature selection is performed during tree construction to separate the data by using the Gini index for each feature.

#### Logistic regression

LR is the best choice when the data involves one or two variables. it is a statistical model and it is the best choice for the binary class classification problem. It also gives better results on the categorical variable. LR is the best learning model because it uses a regression model for the prediction of the probability of class members [36]. The estimation of the probability of an association among one or more independent variables and a categorical dependent variable uses a logistic function for the probability estimation. A logistic function or logistic curve is a common “S” shaped or sigmoid curve, as in the below equation

$$\begin{array}{c} f\left(x\right)=\frac{L}{1+e^{-m(v-v_{o})}} \end{array}$$ (3)

In this study, the LR model was used since it is good for binary classification problems and also works well with numeric data.

#### Gaussian Naive Bayes

GNB is based on the Bayes theorem. When developing its algorithm, it considers certain assumptions, such as assuming that all of the features in the model are independent [37]. It is used for object classification using uniformly distributed data. It is also known as the GNB classifier because of these features. It can be calculated from the following formula

$$\begin{array}{c} P\left(c|x\right)=\frac{P(c|x)P(c)}{p(x)} \end{array}$$ (4)

$$\begin{array}{c} P\left(c|x\right)=P\left(x_{1}|x\right)*\ldots .,P\left(x_{1}|x\right)*P\left(c\right) \end{array}$$ (5)

#### Stochastic gradient decent

The SGD working principle is based on the LR and SVM working principles. The LR convex loss function availability in the SGD makes it a strong classifier. For the multiclass classification problems, SGD is the best option [38]. In the one-versus-all method, SGD aggregates multiple classifiers. The efficiency with which SGD handles large datasets is one of its strengths. It just uses one example per iteration. As the regression method is used in the SGD, it is quite simple to understand and easy to implement. In terms of feature scaling, SGD has a high sensitivity value so, it needs to be accurately valued for better results.

### Convolutional neural networks

For feature engineering, the CNN model is made up of four layers such as a max-pooling layer, an embedding layer, a flatten layer, and a 1D convolutional layer. The embedding layer uses 20 features from the Student academic performance dataset with a vocabulary size of 20,000, with an input length of 20 and 300 output dimensions. After the embedding layer, there is a layer called the 1D convolutional layer that is used with the 5000 filters, a ReLU as an activation function, and a 2×2 kernel size. The 1D convolutional layer is followed by a max-pooling layer. this is implemented with the pool size of 2×2 for mapping the important features from the 1D convolution output. In the end, a flattened layer is used. The function of this layer is to transform the output into a 1-dimension array for the machine learning models because the learning models work well on 1-dimension data [39].

Suppose that the student academic performance dataset *X* is a tuple set *fs*_*i*_, *tc*_*i*_), where *fs* is the feature set,*tc* is the target class column, and *I* is the index of the tuple; then transform the training set in the embedding layer into the required input format as follows

$$\begin{array}{c} EL=embedding\_layer(Vs,Os,I) \end{array}$$ (6)

$$\begin{array}{c} EOs=EL(fs) \end{array}$$ (7)

where *EO*_*s*_ shows the embedding layer output that can develop the convolutional layer inputs, and embedding layer EL comprises three different parameters; one vocabulary size Vs, two output dimensions, and three input lengths.

The size of the input to the model is directly related to the vocabulary size. this study uses the fixed vocabulary size of 20,000, which means that the model can take the inputs from the size range of 0 to 20,000. Secondly, when the data passes through the embedding layer its output dimension is 300, which means that the output dimension parameter *Os* has a value of 300. The third most important parameter is the input length *I*, which shows the number of features in the student academic performance dataset which is 20. the data is processed in the input layer. This input data creates the output for further processing by the CNN model. The input dimensions of the embedding layer are *EOs* = (*None*, 32, 300).

$$\begin{array}{c} 1D-Convs=CNN(F,Ks,AF)\leftarrow EOs \end{array}$$ (8)

where 1D convolutional layers output is represented by the 1D-Convs.

The 1D convolutional layer output is extracted from the embedding layer. In this study, we used the 5000 filters i.e. *F* = 500, in the CNN layer and the kernel size is *Ks* = 2 × 2. ReLU has used the activation function which sets the negative values to zero in the 1*D* − *Convs* output matrix while the other remains the same.

$$\begin{array}{c} f(x)=max(0,E)s \end{array}$$ (9)

The max-pooling layer maps the significant features from the CNN. For the feature map, a 2×2 pool is used. Here Fmap shows the features obtained after the pooling, *Ps* = 2 represents the pooling window size and *S* − 2 is the stride:

$$\begin{array}{c} Cf=Fmap=\lfloor \left(1-P_{s}\right)/S\rfloor +1 \end{array}$$ (10)

To convert the 3D data into a 1D flattened layer is used. The reason for using the 1D data is that the machine learning models work well with the 1D data. By applying these 25,000 features are obtained for the ML model training.

### Evaluation metrics

Testing data is used to determine whether the system has been properly trained. For the testing proposes there are a lot of evaluation parameters available. Accuracy, Precision, recall, and F1 score techniques are used to validate the proposed model’s results where TP is a true positive, TN is a true negative, FP is a false positive and FN is a false negative.

Accuracy is used to measure and evaluate target class correctness. This score has a value range from 0 to 1.

$$\begin{array}{c} Accuracy=\frac{\text{Number}\;\text{of}\;\text{correct}\;\text{predictions}}{\text{Total}\;\text{number}\;\text{of}\;\text{predictions}} \end{array}$$ (11)

By using the following equation the accuracy for the binary class classification can be computed:

$$\begin{array}{c} Accuracy=\frac{TP+TN}{TP+TN+FP+FN} \end{array}$$ (12)

where TP is a true positive, TN is a true negative, FP is a false positive and FN is a false negative. Precision is used to calculate and measure classifier accuracy. Precision is measured by dividing the number of TP by the total of TP and FN. It can be calculated from the following formula:

$$\begin{array}{c} Precision=\frac{TP}{TP+FP} \end{array}$$ (13)

Recall of a classifier is used to determine its completeness. Recall may be calculated by dividing the number of TP by the total of TP and FN.

$$\begin{array}{c} Recall=\frac{TP}{TP+FN} \end{array}$$ (14)

The balance between recall and precision is represented by the F1 score. In other words, the F1-score is the harmonic mean of precision and recall. It has a value between 1 and 0.

$$\begin{array}{c} F1-Score=2\times \frac{Precision\times Recall}{Precision+Recall} \end{array}$$ (15)

## Experiments and results

For the performance evaluation of the proposed approach, a series of experiments are carried out in this study. The machine used in the experiments is an Intel Corei7 7th generation machine with a Windows 10 operating system. python is used as an environment for the implementation of the proposed system and machine learning and deep learning models. In Python, the Keras, Sci-kit learn, and TensorFlow, frameworks are used for the implementation. Experiments are performed in three scenarios: using the original feature set from the student academic performance dataset, using SMOTE features, and CNN features.

### Performance of machine learning models using original features

Firstly, for the student academic performance prediction, experiments are carried out using the original feature set from the student’s academic performance dataset, and the results using the original features are shown in Table 3. From the results, it is clear that the tree-based ensemble model ETC achieved the highest accuracy score of 0.887 and outperformed the other machine learning models used in this study on the original feature set. Other tree-based methods like RF reach a similar accuracy value of 0.867. The ensemble of linear models, such as GBM and GNB also perform better in terms of accuracy score, with 0.836 and 0.857, respectively. However, the linear model such as LR is the least performing individual linear model, with a 0.765 accuracy score.

**Table 3:** Performance of machine learning models using original features.

Model	Accuracy	Class	Precision	Recall	F1 score
RF	0.867	Pass	0.89	0.40	0.55
		Fail	0.87	0.99	0.92
		Weighted Avg.	0.87	0.87	0.85
ETC	0.887	Pass	0.85	0.55	0.67
		Fail	0.89	0.97	0.93
		Weighted Avg.	0.88	0.89	0.88
GBM	0.836	Pass	0.67	0.40	0.50
		Fail	0.86	0.95	0.90
		Weighted Avg.	0.82	0.84	0.82
LR	0.765	Pass	0.00	0.00	0.00
		Fail	0.79	0.96	0.87
		Weighted Avg.	0.63	0.77	0.69
GNB	0.857	Pass	0.88	0.35	0.50
		Fail	0.86	0.99	0.92
		Weighted Avg.	0.86	0.86	0.83
SGD	0.795	Pass	0.00	0.00	0.00
		Fail	0.80	1.00	0.89
		Weighted Avg.	0.63	0.80	0.71

**Fig 4:**
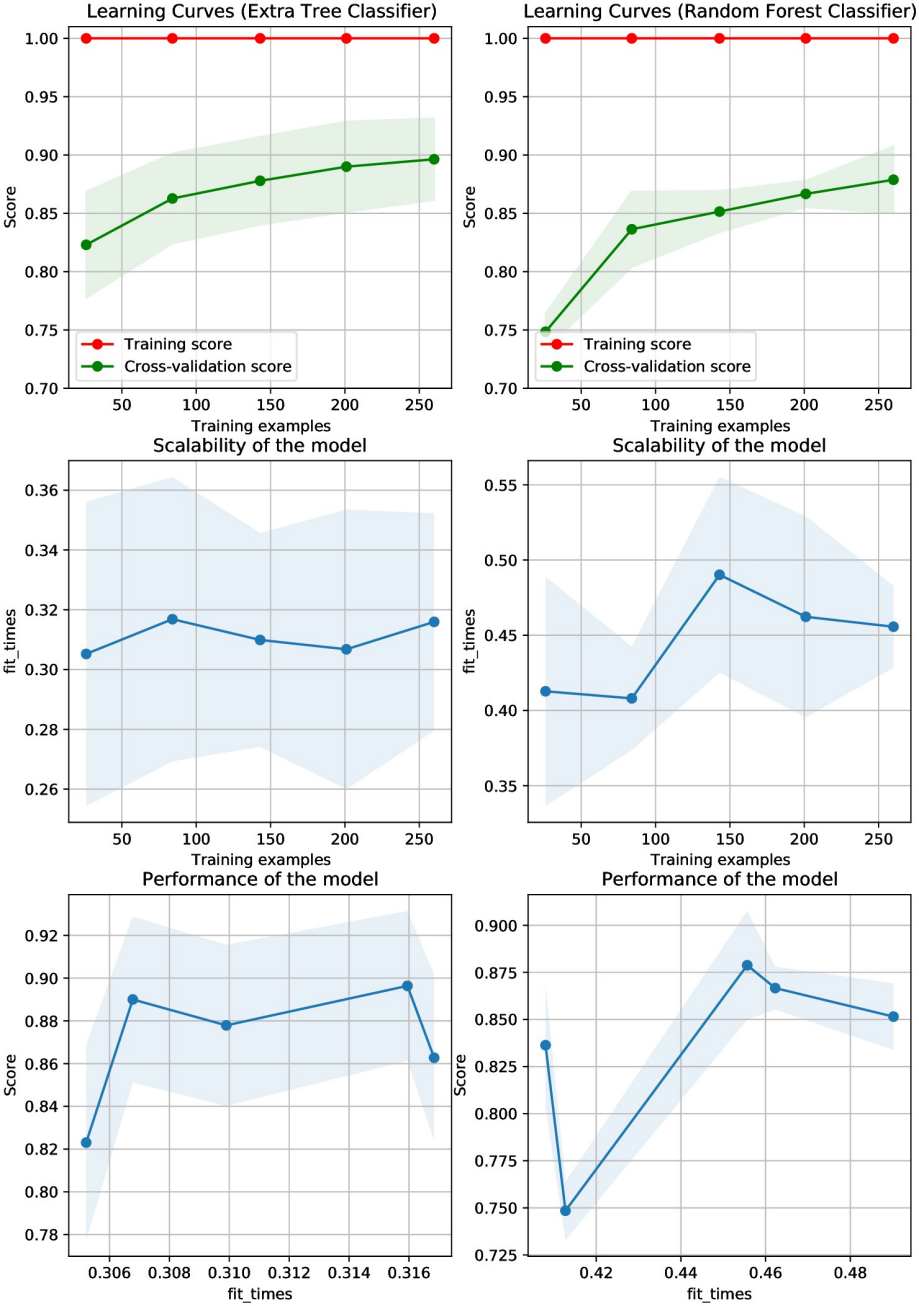
Learning curves of models using the original dataset.

When compared to linear models, the tree-based model ETC performs better. Also, the performance of the other liner model with the large feature set is good. On the other hand, tree-based models can be useful even when the feature set is limited. Because the dataset used in the study only has 326 instances, ETC performs effectively with a small feature set. Fig 4 shows the learning curves and scalability of the models. Despite ETC’s improved performance, its accuracy does not satisfy the criteria of students’ academic performance accuracy measuring and must be improved further. Because the dataset used in the study is significantly imbalanced, SMOTE is used and further experiments are performed.

### Performance of machine learning models using SMOTE features

**Fig 5:**
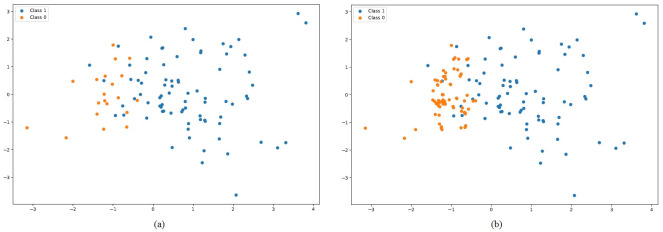
Data samples for both classes, (**a**) Before SMOTE, and (**b**) After SMOTE.

In the second set of experiments, the SMOTE features are used to examine the performance of machine learning and the proposed ensemble model. Fig 5 shows the data sample before SMOTE and after applying SMOTE. For both classes, it can be seen that both classes become almost equal when SMOTE is applied. The data balancing helps the model train better and the probability of model overfitting on the majority class is minimized.

The results of the classifiers using the SMOTE-balanced dataset are shown in Table 4. The results show that the tree-based ensemble model RF beats other models with an accuracy value of 0.905. Other tree-based models, such as the ETC and GBM classifier, achieved comparable accuracy values of 0.899. With 0.672 accuracy, linear models such as LR is the lowest performer with an accuracy score of 0.672. However, when compared to individual linear models, the ensemble of linear models performs better.

**Table 4:** Performance of machine learning models using SMOTE-balanced dataset.

Model	Accuracy	Class	Precision	Recall	F1 score
RF	0.905	Pass	0.90	0.93	0.91
		Fail	0.92	0.88	0.90
		Weighted Avg.	0.91	0.91	0.91
ETC	0.899	Pass	0.90	0.92	0.91
		Fail	0.90	0.88	0.89
		Weighted Avg.	0.90	0.90	0.90
GBM	0.899	Pass	0.89	0.93	0.91
		Fail	0.92	0.87	0.89
		Weighted Avg.	0.90	0.90	0.90
LR	0.672	Pass	0.70	0.68	0.69
		Fail	0.65	0.67	0.66
		Weighted Avg.	0.67	0.67	0.67
GNB	0.735	Pass	0.76	0.74	0.75
		Fail	0.71	0.73	0.72
		Weighted Avg.	0.74	0.74	0.74
SGD	0.691	Pass	0.68	0.77	0.73
		Fail	0.70	0.60	0.65
		Weighted Avg.	0.69	0.69	0.69

**Fig 6:**
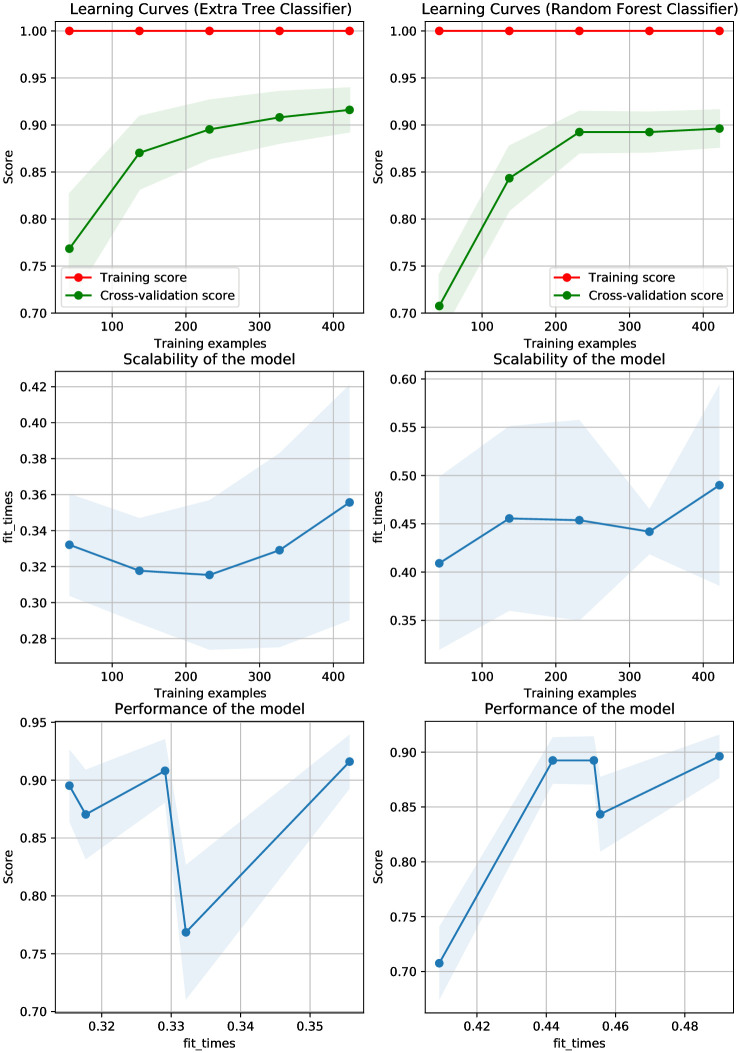
Learning curves of models on the SMOTE-balanced dataset.

Linear models typically perform well with a large feature set and the performance of the tree-based model RF is better than linear models. On the other hand, Tree-based models can be useful even when features are limited. Fig 6 shows the learning curves for different models using the balanced dataset. For further analysis of the machine learning models’ performance, further experiments are carried out using the CNN-extracted features.

### Performance of machine learning models using CNN features

The third set of experiments is carried out using CNN features to examine the performance of machine learning and the proposed model to achieve improved accuracy for the measurement of student academic performance. Experimental results are shown in Table 5. Results of the machine learning models using the CNN features show that the proposed tree-based model ETC shows better results as compared to the other learning models with the highest accuracy of 0.999. It indicates an improvement of 10.0% in the performance of ETC than its performance with the SMOTE features and an 11.2% improvement in the performance of the proposed ETC with the original features. Similarly, the other tree-based model RF archives an accuracy value of 0.985. It also achieves better results with CNN features than with the original feature set. LR achieves 0.872 accuracy while GNB obtains a 0.835 accuracy score representing that there is an improvement of 10% than SMOTE and a 2% drop in accuracy than the original features.

**Table 5:** Performance of models using CNN features.

Model	Accuracy	Class	Precision	Recall	F1 score
RF	0.985	Pass	0.90	0.93	0.91
		Fail	0.92	0.88	0.90
		Weighted Avg.	0.91	0.91	0.91
ETC	0.999	Pass	0.90	0.92	0.91
		Fail	0.90	0.88	0.89
		Weighted Avg.	0.90	0.90	0.90
GBM	0.969	Pass	0.89	0.93	0.91
		Fail	0.92	0.87	0.89
		Weighted Avg.	0.90	0.90	0.90
LR	0.872	Pass	0.70	0.68	0.69
		Fail	0.65	0.67	0.66
		Weighted Avg.	0.67	0.67	0.67
GNB	0.835	Pass	0.76	0.74	0.75
		Fail	0.71	0.73	0.72
		Weighted Avg.	0.74	0.74	0.74
SGD	0.891	Pass	0.68	0.77	0.73
		Fail	0.70	0.60	0.65
		Weighted Avg.	0.69	0.69	0.69

The aim of using CNN model features is to expand the feature set, which is expected to boost linear model accuracy. Machine learning models are trained and tested using CNN-extracted features for this purpose. When CNN is used for feature extraction, the accuracy of models improves significantly when they are used with convoluted features from CNN, which is attributed to the increase in the number of features.

**Fig 7:**
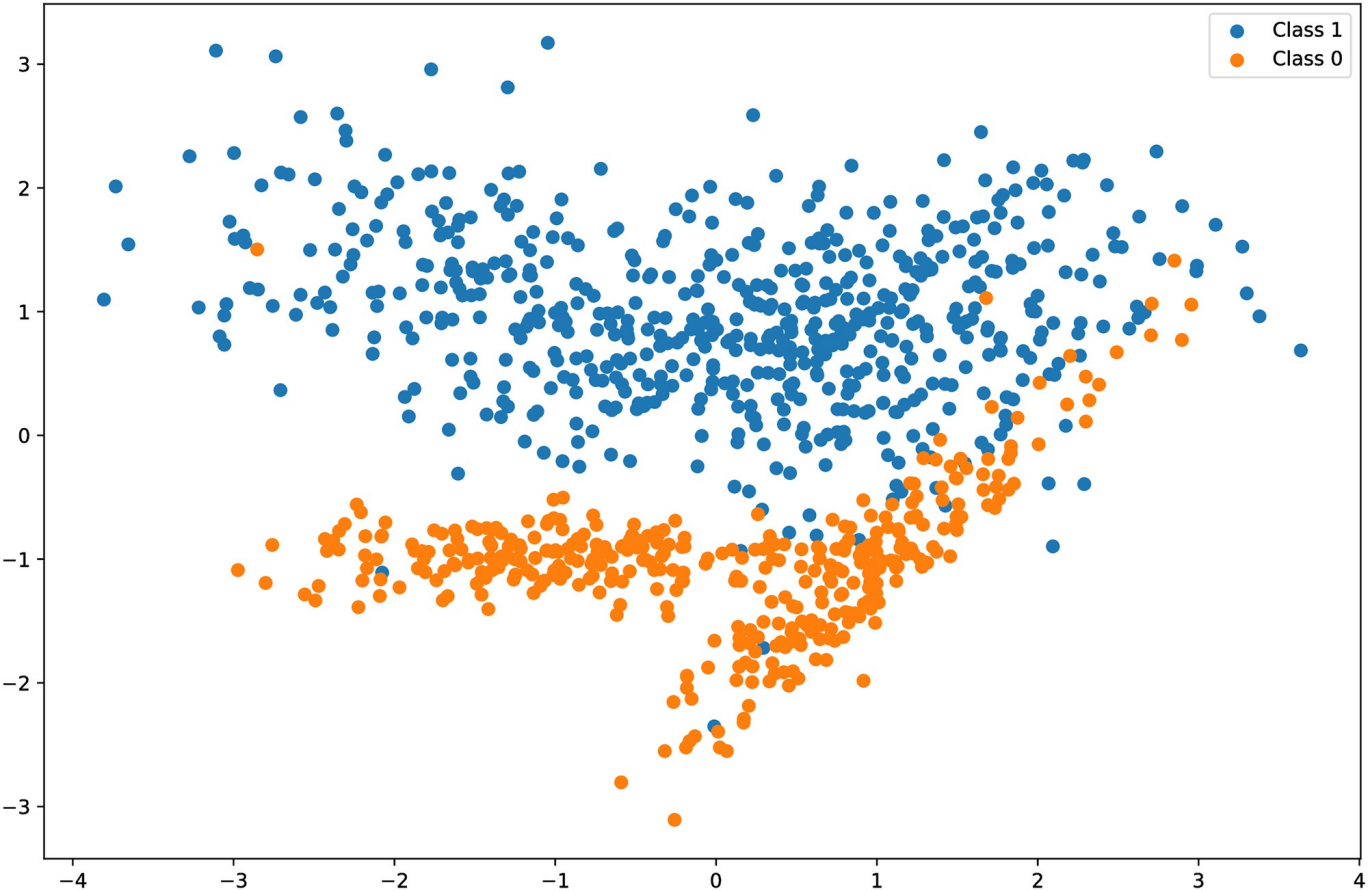
Convolution features obtained using CNN model.

**Fig 8:**
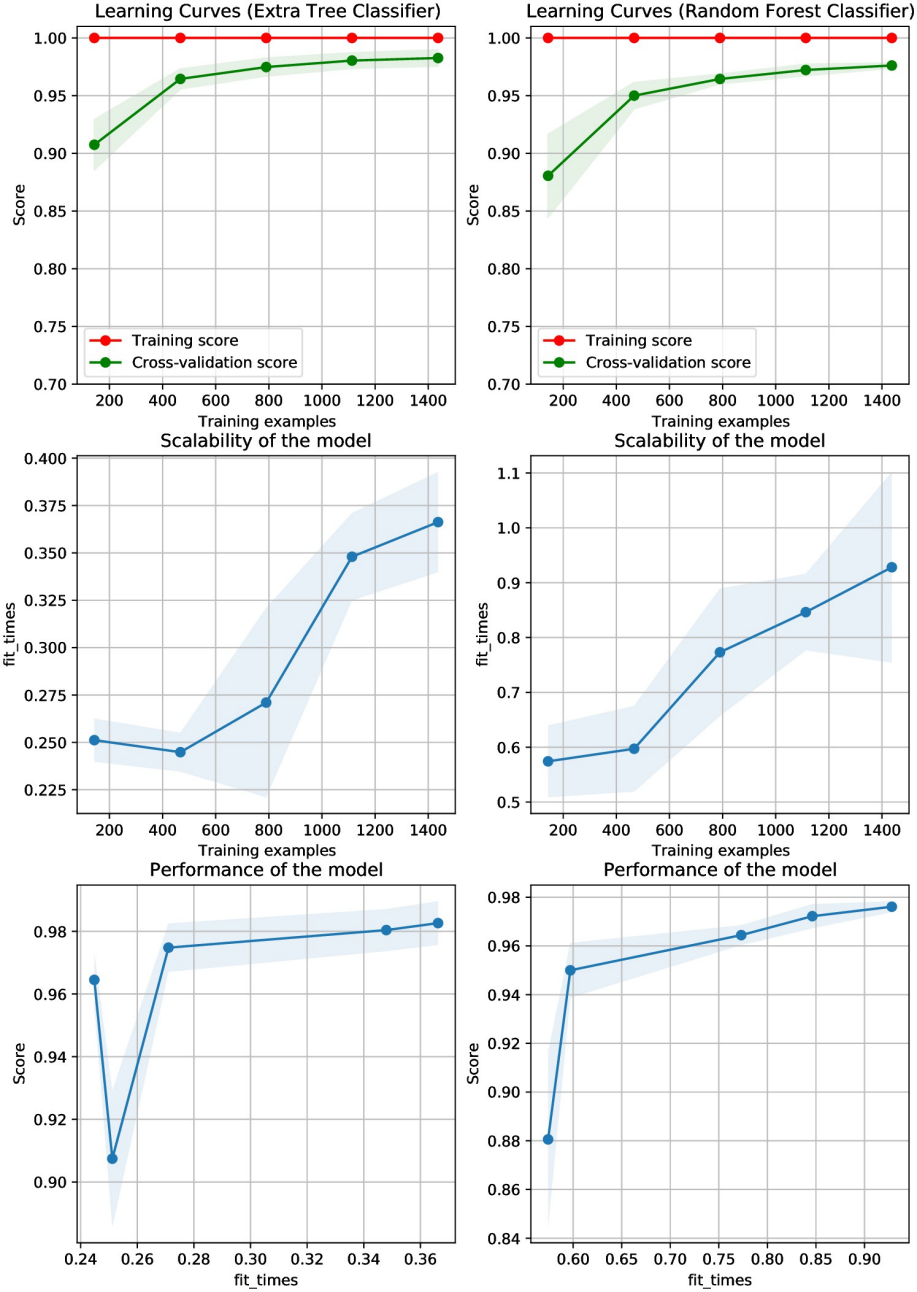
Learning curves of models using CNN-extracted features.

Fig 7 shows the CNN features obtained using the CNN model which indicates that the distribution of the feature space for two classes provides better features for models’ training which improves their performance. Consequently, the learning curves and performance of the models are improved as shown in Fig 8. Because CNN model features are highly correlated with the target class and make data linearly separable, linear models outperform other models.

Deep convolutional features in educational data analysis are crucial for educators and administrators. It involves visualizing these features, aligning them with educational theories, identifying important factors affecting student performance, analyzing temporal trends, ranking feature importance, establishing a feedback loop with educators, and addressing ethical considerations. This interpretability empowers stakeholders to make informed decisions, tailor interventions, and enhance the educational experience for students.

### Performance comparison with existing studies

Results are compared with several approaches from the existing literature for performance validation and robustness analysis. To achieve significant results, this research used various machine learning or deep learning models. Studies [40, 41] made use of bi-directional LSTM models for predicting confused students. [41] additionally leverages the feature selection approach to enhance the model’s performance. An ETC model is adopted in [42] for the same purpose. The current study employs deep convolutional features with ETC for student academic performance prediction using features from student academic activities, video interactions, and academic performance. Comparison results given in Table 6 show that the proposed approach produces better results. In the end, Table 7 shows all the acronyms used in this study.

**Table 6:** Performance comparison with state-of-the-art studies.

Reference	Year	Approach	Accuracy
[40]	2017	Bidirectional LSTM	0.73
[43]	2019	CF Bidirectional LSTM	0.75
[41]	2020	Bidirectional LSTM	0.88
[42]	2022	Extra Tree Classifier	0.66
Proposed	2022	CNN features and Extra Tree Classifier	0.99

**Table 7:** Acronym table with description.

Acronym	Description
ADASYN	Adaptive synthetic resampling
AI	Artificial Intelligence
ANN	Artificial neural network
BilSTM	Bidirectional Long Short-Term Memory
CGAN	conditional generative adversarial network
CNN	Convolutional Neural Network
EEG	Electroencephalogram
EDM	Educational data mining
ETC	Extra tree classifier
FN	False Negative
FP	False Positive
GBM	Gradient boosting machine
GNB	Gaussian Naive Bayes
HEIs	Higher education institutions
ICT	Information and communication technology
KNN	k nearest neighbor
LMS	Learning management system
LR	Logistic regression
MOOC	Massive open online course
PBF	Probability based features
RF	Random forest
SGD	Stochastic gradient descent
SMOTE	Synthetic minority oversampling technique
TN	True Negative
TP	True Positive

### Challenges and opportunities

Implementing an AI-based strategy in actual educational institutions entails a number of challenges and opportunities, taking into consideration various elements.

The challenges are the following:

- AI models can be computationally intensive, requiring substantial hardware and infrastructure, which may strain the IT resources of some institutions.
- Access to high-quality educational data, especially in smaller or less well-funded institutions, can be limited.
- Educators may need training to effectively use AI-driven tools and integrate them into their teaching methods.
- There’s a risk of students becoming overly reliant on AI for learning, potentially reducing their independent problem-solving skills.
- Measuring student engagement accurately through AI may require a combination of behavioral data, surveys, and qualitative assessments.

Opportunities are the following:

- AI can tailor learning experiences to individual students, helping them progress at their own pace and addressing their specific needs.
- AI can detect early warning signs of academic or behavioral issues, enabling timely intervention to prevent students from falling behind.
- AI-powered educational tools have the potential to reach a broader audience, including remote and underserved communities.
- AI can assist educators by providing them with data-driven insights, reducing administrative work, and allowing them to focus on more meaningful teaching activities.

### Future research directions

Future research in the domain of AI in education can explore various promising directions to enhance its effectiveness and impact. Here are some potential areas for future research:

- Investigate advanced AI algorithms for creating highly personalized learning paths for students. This includes dynamically adapting content, assessments, and teaching methods based on individual progress and needs.
- Develop AI-driven systems capable of providing real-time interventions for students who are struggling. These interventions could include targeted feedback, additional resources, or suggestions for alternative learning strategies.
- Explore AI techniques to enhance student engagement in online and hybrid learning environments. This might involve developing AI-driven gamification strategies, interactive content, or intelligent virtual tutors.
- Research ethical considerations in AI-driven education, including bias mitigation, transparency, and fairness. Develop guidelines and best practices to ensure responsible AI use in the educational context.
- Utilize NLP techniques to develop AI-powered chatbots or virtual teaching assistants capable of answering student queries, providing guidance, and facilitating discussions in real-time.
- To improve the generalizability and scalability of the proposed approach, we intend to experiment with larger and more diverse datasets in the future.

## Conclusion

The motivation of this research work is to develop a framework that accurately predicts students’ academic performance and helps to take preventive measures to reduce the risk of student dropout. The proposed approach aims at increasing the accuracy while minimizing the prediction error for student academic performance. To this end, deep convoluted features are obtained from a customized CNN and used to train the machine learning models. SMOTE-balanced data shows better performance than the original features. However, experimental results indicate that using convolutional features tends to obtain higher accuracy than the original features. Moreover, the machine learning classifier ETC shows the best performance than other models. Performance comparison with state-of-the-art studies shows the superior performance of the proposed approach. Again the higher accuracy as compared to other approaches shows the effectiveness of this framework. One shortcoming of this study is the limited data samples which make it difficult to obtain generalizability. We intend to increase the dataset size and perform further experiments in the future.
